# Proposed Special Service (Cholera) Corps

**Published:** 1914-10-17

**Authors:** A. White Robertson

**Affiliations:** Hon. Sec. The New Laboratory, 67 Margaret Street, Cavendish Square, W.


*To the Editor of* The Hospital.

### Proposed Special Service (Cholera) Corps.

*To the Editor of* The Hospital.

Sir,—As there seems to be no call for the services of this Corps in the immediate future, I have, after correspondence with the War Office and British Red Cross Society, to inform the large number of medical men and nurses who have applied for special training that the project will now be dropped, leaving its members free to accept other appointments.—Yours faithfully,

A. White Robertson, L.R.C.P. & S. Edin.,
Hon. Sec.

The New Laboratory,
67 Margaret Street, Cavendish Square, W.

[The project was referred to in The Hospital of August 29, p. 592.]

